# Global convergence of COVID-19 basic reproduction number and estimation from early-time SIR dynamics

**DOI:** 10.1371/journal.pone.0239800

**Published:** 2020-09-24

**Authors:** Gabriel G. Katul, Assaad Mrad, Sara Bonetti, Gabriele Manoli, Anthony J. Parolari

**Affiliations:** 1 Nicholas School of the Environment, Duke University, Durham, NC, United States of America; 2 Department of Civil and Environmental Engineering, Duke University, Durham, NC, United States of America; 3 Department of Environmental Systems Science, ETH Zürich, Zürich, Switzerland; 4 Bartlett School of Environment, Energy and Resources, University College London, London, United Kingdom; 5 Department of Civil, Environmental and Geomatic Engineering, University College London, London, United Kingdom; 6 Department of Civil, Construction, and Environmental Engineering, Marquette University, Milwaukee, Wisconsin, United States of America; Frankfurt Institute for Advanced Studies, GERMANY

## Abstract

The SIR (‘susceptible-infectious-recovered’) formulation is used to uncover the generic spread mechanisms observed by COVID-19 dynamics globally, especially in the early phases of infectious spread. During this early period, potential controls were not effectively put in place or enforced in many countries. Hence, the early phases of COVID-19 spread in countries where controls were weak offer a unique perspective on the ensemble-behavior of COVID-19 basic reproduction number *R*_*o*_ inferred from SIR formulation. The work here shows that there is global convergence (i.e., across many nations) to an uncontrolled *R*_*o*_ = 4.5 that describes the early time spread of COVID-19. This value is in agreement with independent estimates from other sources reviewed here and adds to the growing consensus that the early estimate of *R*_*o*_ = 2.2 adopted by the World Health Organization is low. A reconciliation between power-law and exponential growth predictions is also featured within the confines of the SIR formulation. The effects of testing ramp-up and the role of ‘super-spreaders’ on the inference of *R*_*o*_ are analyzed using idealized scenarios. Implications for evaluating potential control strategies from this uncontrolled *R*_*o*_ are briefly discussed in the context of the maximum possible infected fraction of the population (needed to assess health care capacity) and mortality (especially in the USA given diverging projections). Model results indicate that if intervention measures still result in *R*_*o*_ > 2.7 within 44 days after first infection, intervention is unlikely to be effective in general for COVID-19.

## Introduction

A heated dispute about the effectiveness versus risk of smallpox inoculation was playing out in eighteenth-century France, which was to launch the use of mathematical models in epidemiology. This dispute moved inoculation from the domain of philosophy, religion, and disjointed trials plagued by high uncertainty into a debate about mathematical models—put forth by Daniel Bernoulli (in 1766) and Jean-Baptiste le Rond D’Alembert (in 1761), both dealing with competing risks of death and interpretation of trials [1]. Since then, the mathematical description of infectious diseases continues to draw significant attention from researchers and practitioners in governments and health agencies alike. Even news agencies are now seeking out explanations to models so as to offer advice and clarity to their audiences during the (near-continuous) coverage of the spread of COVID-19 [2]. The prospect of using mathematical models in conjunction with data is succinctly summarized by the Nobel laureate Ronald Ross, whose 1916 abstract [3] continues to enlighten the role of mathematics in epidemiology today. A quotation from this abstract below, which foreshadows the requirements and challenges for mathematical models to describe emerging epidemics such as COVID-19 [4, 5], needs no further elaboration:

*It is somewhat surprising that so little mathematical work should have been done on the subject of epidemics, and, indeed, on the distribution of diseases in general. Not only is the theme of immediate importance to humanity, but it is one which is fundamentally connected with numbers, while vast masses of statistics have long been awaiting proper examination. But, more than this, many and indeed the principal problems of epidemiology on which preventive measures largely depend, such as the rate of infection, the frequency of outbreaks, and the loss of immunity, can scarcely ever be resolved by any other methods than those of mathematical analysis*.

The classic susceptible-infectious-recovered (SIR) paradigm, initiated in the late 1920s [6], now provides a mathematical framework that describes the core transmission dynamics of a wide range of human diseases [7–12], including COVID-19 [13]. A key parameter in the SIR paradigm is the basic reproduction number (*R*_*o*_). The *R*_*o*_ is defined by the average number of secondary cases arising from a typical primary case in an entirely susceptible population of size *S*_*o*_ [14– 16]. The usefulness of *R*_*o*_ and uncertainty in its estimation are not a subject of debate, as reviewed elsewhere [17], and therefore are not further discussed here.

**Fig 1:**
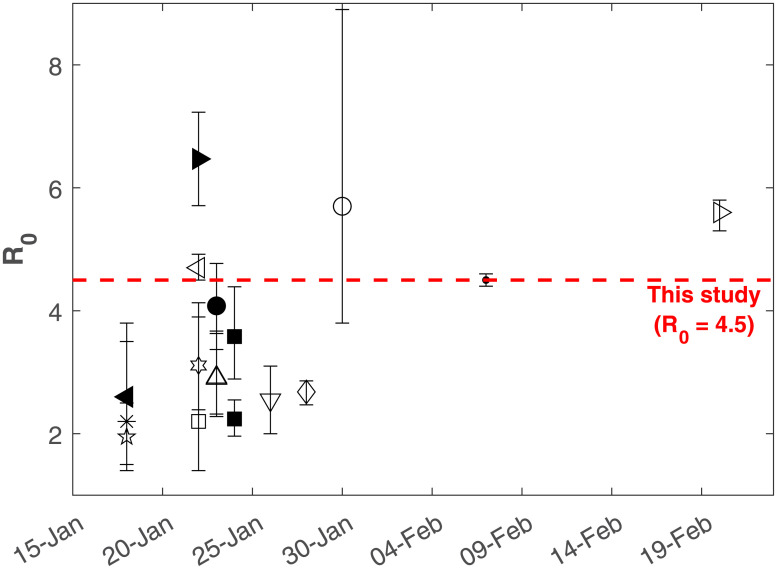
Timeline of the COVID-19 *R*_*o*_ estimates. Symbols represent studies listed in Appendix (Table 1) while the red dashed line marks *R*_*o*_ = 4.5 derived from this study. An initial *R*_*o*_ = 2.2 was initially adopted by the World Health Organization (WHO).

The *R*_*o*_ for COVID-19 and other diseases is commonly estimated directly from case data or by fitting the SIR model or one of its many variants to the data [14, 18–22]. Unsurprisingly, the *R*_*o*_ estimates often exhibit large uncertainty. For COVID-19, mean estimated *R*_*o*_ ranges between 1.95 and 6.47, with corresponding error estimates giving upper and lower bounds of 1.4 and 8.9 (Fig 1). For a given virus, such variability in *R*_*o*_ is attributed to local spatio-temporal variability in public health resources, interventions, and how individuals in a population interact, among others, as well as the estimation method used [15, 17]. Due to this local variability, it is commonly held that *R*_*o*_ is a site-specific parameter that cannot be directly transferred between sites. The rapid availability of global data for COVID-19 has allowed an unprecedented comparison across a diverse array of populations, which is absent from the prior literature on *R*_*o*_ estimation.

In the analysis herein, the SIR model is used to uncover generic spread mechanisms observed by COVID-19 dynamics globally, especially in the early phases of infectious spread. During this early period, potential controls were not effectively put in place or enforced in many countries around the world despite early warning signals from China, Iran, and later on, Italy. Hence, the early phases of COVID-19 spread in many countries where controls were weak offer a unique perspective on the ensemble-behavior of COVID-19 *R*_*o*_. The analysis shows that there is global convergence (i.e. across many nations) to an uncontrolled *R*_*o*_ = 4.5 for COVID-19 describing early times spread from the SIR model. This value is compared to a number of published *R*_*o*_ estimates for COVID-19 with a timeline summary featured in Fig 1. These published estimates along with the methods used to infer *R*_*o*_, the published uncertainty, and the original data source are provided in the Appendix.

Clearly, such wide ranging values of *R*_*o*_ in Fig 1 motivate further analysis of *R*_*o*_ variability across populations, the objective of this analysis. Other aspects that are considered in the estimation of *R*_*o*_ from SIR are the effects of ramp-up in testing at the early phases of disease spread and the role of super-spreaders. These two effects are briefly discussed using idealized scenarios and model calculations. The implications for evaluating potential control strategies from this uncontrolled *R*_*o*_ are considered in the context of mortality and maximum infections.

## Theory

### Definitions and nomenclature

Mathematical models of disease spread assume that a population within a compartment (e.g., city, region, country) can be subdivided into a set of distinct classes [11]. The SIR model classifies individuals in the compartment as one of three classes: susceptible (*S*), infectious (*I*), and recovered or removed (*R*). Infectious individuals spread the disease to susceptible individuals and remain in the infectious class for a given period of time known as the infectious period before moving into the recovered (or removed) class. Individuals in the recovered class are assumed to be immune for an extended period (or removed from the population). For the total population *N* = *S* + *I* + *R*, the dynamical system describing the SIR equations are given as

$$\begin{array}{cc} \frac{dS}{dt} & =-(\beta \frac{I}{N})S \end{array}$$ (1)

$$\begin{array}{cc} \frac{dI}{dt} & =+(\beta \frac{I}{N})S-\gamma I \end{array}$$ (2)

$$\begin{array}{cc} \frac{dR}{dt} & =+\gamma I, \end{array}$$ (3)

where *β*(*I*/*N*) is known as the force of infection and coefficients *β* and *γ* must be externally supplied. Moreover, this system requires the specification of three initial conditions at time *t* = 0, *S*(0), *I*(0), and *R*(0). For COVID-19, it is assumed that *R*(0) = 0 and *I*(0)≪*S*(0) at the initial outbreak. For the initial conditions selected here, *N* = *S*(0) + *I*(0) + *R*(0) ≈ *S*(0), which is labeled *S*_*o*_ for notational simplicity and consistency with the SIR literature. The basis of the latter assumption is that the number of deceased individuals is ≪*S*_*o*_. The dynamical system in Eqs (1), (2), (3) has only one equilibrium point: *I* = 0 for any *S* > 0, which is a disease-free stable equilibrium (i.e. as *t* → ∞, *I*(*t*) → 0).

### SIR model assumptions

The SIR model makes a number of assumptions, including a closed system with no changes in natural births or natural deaths occurring during the short-lived outbreak. The infection is assumed to have negligible latent period so that an individual becomes infectious when infected. For this reason, the SIR model might underestimate *R*_*o*_ but the uncertainty surrounding the incubation period possibly precludes any advantage of adding a ‘incubation’ compartment to the SIR model [23] at this stage. Recovering from infection is also assumed to confer long-term immunity, yet to be verified for COVID-19.

The most objectionable assumption in SIR dynamics is the use of the so-called ‘mass-action’ principle. As with all compartment models, mass action assumes that the rate of encounter between *I* and *S* is proportional to their product. For this assumption to hold, it requires that members of *I* and *S* be uniformly distributed in the space of the compartment [24]. Individuals—unlike molecules in an ideal solution within a closed container—do not mix homogeneously. Nonetheless, the use of the mass action principle serves as one reference to estimate *R*_*o*_ in a consistent manner across differing countries using the SIR framework. The presence of super-spreaders on *R*_*o*_ estimates using spatially-extended analysis of SIR is to be discussed later on.

The parameters *γ* and *β* encode the main properties of the epidemics and the population response to it. The *γ* = 1/*D* is generally interpreted as the inverse of the mean recovery time *D*. The *D* varies with the nature of the disease and the recovery from it, which depends on the medical facilities and resources available. For COVID-19, the best information on the speed of recovery comes from a World Health Organization study examining more than 55,000 cases in China [25]. They found that for mild illness, the time from the onset of symptoms to natural recovery is, on average, 14 days. This estimate was also supported in other published studies (e.g., [26]), though as much as 6-8 weeks were recorded for severe infections. Because *I* is dominated by mild cases thus far, *D* = 14 d is selected here.

With this assumption, the remaining model parameter *β* must be determined empirically or from separate studies. The *β* reflects the multiplicative effect of two factors: (1) the transmissibility of the infectious disease (= *T*_*r*_) or the probability of disease transmission after an encounter between a susceptible and an infected and (2) the number of contacts per unit time *k* each infected individual has with susceptibles. Hence, *β* = *kT*_*r*_. Factors such as hand-washing and sanitizing or wearing masks reduce *T*_*r*_ whereas social distancing, self-isolation, and closure of public or crowded spaces reduce *k*. From Eq (2), it is evident that *dI*/*dt* will be positive (outbreak) or negative (epidemic contained) depending on the sign of (*β*(*S*/*S*_*o*_) − *γ*), which is one of the main reasons the basic reproduction number is sought.

### The basic reproduction number *R*_*o*_

As earlier stated, the average rate of recovery is set to *γ* = 1/*D*. Given the value of *D* (in days), the probability that an individual remains infected in an infinitesimal time period *δτ* is 1 − *γ*(*δτ*). Therefore, the probability that this individual remains infected for an amount of time *τ* is lim_*δτ* → 0_(1 − *γδτ*)^*τ*/(*δτ*)^ = exp(−*γτ*). In other words, *τ*, the time that an infected individual remains infected, is exponentially distributed with an average of *D* = 1/*γ*.

In a compartmental model such as the SIR, every individual is initially susceptible and the average number of susceptibles that encounter an infected individual over a period *τ* is simply *βτ*. It follows that the average number of new infections caused by an infected individual, which is the basic reproduction number *R*_*o*_, is given by [27]

$$\begin{array}{c} R_{o}=\beta \;\int_{0}^{\infty }\tau \;p\left(\tau \right)d\tau =\beta \;\gamma \int_{0}^{\infty }\tau e^{-\gamma \tau }d\tau =\frac{\beta }{\gamma }, \end{array}$$ (4)

where the *γ* after the second equality is to normalize *p*(*τ*)*dτ*.

Two assumptions underlying the compartmental SIR model are unrealistic, but a proven correspondence between the compartmental SIR and a Poisson graph SIR model justifies its applicability. In the compartmental (or fully-mixed) SIR model, the recovery times are exponentially distributed and every individual has an equal chance per unit time of encountering all other *S*_*o*_ − 1 individuals [27]. However, COVID-19 recovery times have been determined to concentrate around *D* = 1/*γ* = 14 days. Moreover, infected individuals come in contact with only a handful of other people. But, it was shown that the dynamics of a discrete-time SIR compartmental model (Reed-Frost model) and those of the SIR on a Poisson random graph are equivalent [28]. The Poisson random graph model assumes a constant recovery time and a Poisson distributed degree distribution (i.e., number of contacts for every individual), both more realistic assumptions. It is this correspondence between the SIR model and the random graph that makes the SIR model an appropriate tool to explore the early-time dynamics of COVID-19 spread.

### Early-times dynamics of the SIR system

As common with dynamical systems, non-dimensional variables are preferred in the analysis of the phase-space to be conducted next. Here, *γ* and *S*_*o*_ are obvious choices for normalizing time and population pools. Hence, a dimensionless time t_*_ = *γt* and a dimensionless fraction of individuals *s* = *S*/*S*_*o*_, *i* = *I*/*S*_*o*_, and *r* = *R*/*S*_*o*_ are introduced so that the original SIR system is now

$$\begin{array}{cc} \frac{ds}{dt_{*}} & =-(R_{o}i)s \end{array}$$ (5)

$$\begin{array}{cc} \frac{di}{dt_{*}} & =+(R_{o}i)s-i \end{array}$$ (6)

$$\begin{array}{cc} \frac{dr}{dt_{*}} & =+i. \end{array}$$ (7)

**Fig 2:**
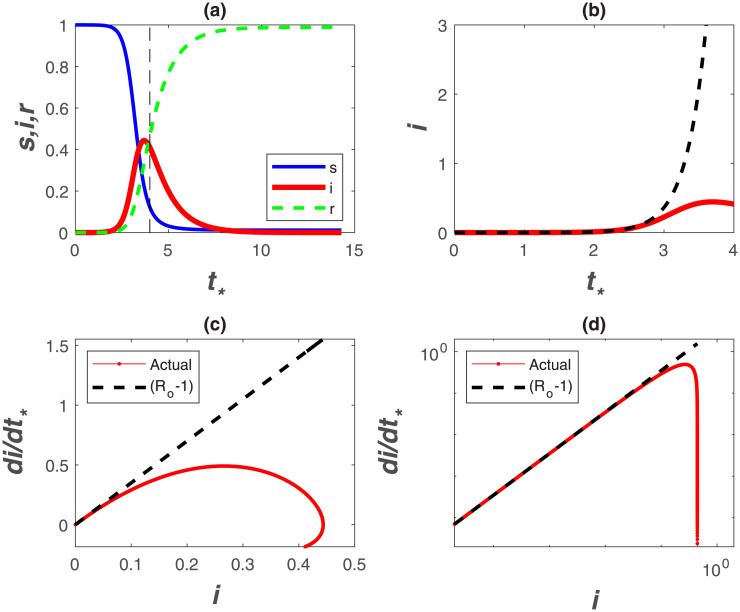
Phase space and temporal trends of the SIR model. (a) *S*(*t*), *I*(*t*), *R*(*t*) normalized by *S*_*o*_ as a function of dimensionless time t_*_ = *γt* with *S*_*o*_ = 100, 000, *γ* = (1/14) d^−1^, and *R*_*o*_ = 4.5. (b) *i* = *I*/*S*_*o*_ in dimensionless time t_*_ = *γt* for early times t_*_ < 1 revealing strictly exponential growth (dashed) and deviations from exponential (SIR solution). (c) *di*/dt_*_ with *i* in linear and (d) double-log representations. The dashed line is (*R*_*o*_ − 1) where *R*_*o*_ = 4.5. Declines from the dashed line reflect the incipient point where *i*(*t*) deviates appreciably from exponential growth. Note how the early-time slope (*R*_*o*_ − 1) is emphasized in the double-log representation.

An illustration of the normalized SIR dynamics during an epidemic is shown in Fig 2a, where *s*, *i*, and *r* are numerically solved when setting *R*_*o*_ = 4.5, *γ* = (1/14) d^−1^ and *S*_*o*_ = 100, 000. For small t_*_(< 1), *s* ≈ 1 as seen from Fig 2a. In this early phase, *s*(≈ 1) can be ‘de-coupled’ from *i* resulting in an autonomous budget for *i* given as

$$\begin{array}{c} \frac{di}{dt_{*}}\approx i(R_{o}-1). \end{array}$$ (8)

When *R*_*o*_ > 1, *di*/dt_*_ > 0 leading to an epidemic or, conversely, a containment of the disease. The solution of Eq (8) is an exponential function *i*(*t*)/*i*(0) = exp[(*R*_*o*_ − 1)t_*_] shown in Fig 2b.

The *R*_*o*_ may be determined by regressing log(*i*) against *t*, and the slope of this regression determines *R*_*o*_ when *γ* can be separately estimated. More sophisticated fitting procedures can also be conducted on sampled *I*(*t*) versus *t*. A major limitation to this exercise is that *I*(*t*) at early times, often determined from reported confirmed cases, is uncertain and depends on testing frequency that may vary in time as *I* increases. An alternative is to regress *di*/dt_*_ upon *i* at early times to detect the highest slope, which can then be used to infer *R*_*o*_. This approach is featured in Fig 2c, which illustrates that the SIR dynamics exhibit rapid deviations from a linear *di*/dt_*_ with *i* set by early times thereby underestimating *R*_*o*_ (for a given *γ*). Evidently, inference of *R*_*o*_ requires estimates of early time slope, which cannot be easily detected in practice.

A non-conventional approach is to present confirmed infection data using a double-log representation of *di*/dt_*_ versus *i*, which is featured in Fig 2d. This presentation has a number of advantages and limitations in the analysis of COVID-19 discussed elsewhere [29]. The main advantage is that the early time slope (= *R*_*o*_ − 1) visually persists over much of the graph. A significant decline in *di*/dt_*_ is also required before ‘registering’ a drop in such a representation. This insensitivity to moderate declines in *di*/dt_*_ from its initial value may be advantageous in *R*_*o*_ estimates. The other main limitation, which is inherent to all such analyses, is shifts in testing frequency at high *i*, and thus the increase in confirmed cases due to expanded testing. It is to be noted that a log-log representation will be more robust to these shifts, because the overall graph will be biased by the initial slope prior to the initialization of expanded testing. Such bias should lead to increases in *di*/dt_*_ versus *i*, not declines from the initial slope (*R*_*o*_ − 1) that can be detected. As later shown, such an increase has been noted in several data sets.

With this representation, it is now shown that initial inaction to COVID-19 across many countries around the globe allowed an ensemble estimate of the uncontrolled *R*_*o*_. Because *R*_*o*_ is likely to be at maximum when no action to COVID-19 are implemented early on, a maximum theoretical ‘boundary-line’ can then be derived to describe the spread of COVID-19 for large *S*_*o*_ (on log-log representation). This boundary-line analysis can then be used as a logical reference to assess whether measures to reduce *β* are effective.

## Results and discussion

### Estimating an early-time *R*_*o*_

**Fig 3:**
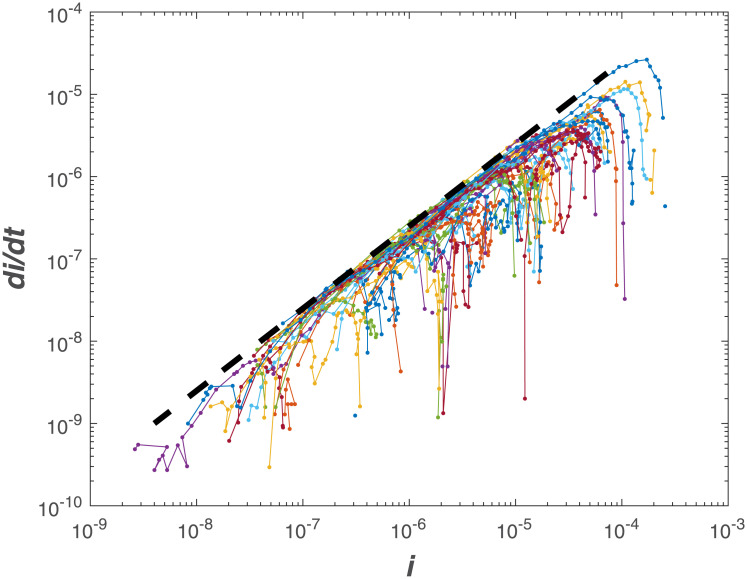
Comparison between *di*/*dt* and *i* for 57 countries. The dashed line is (*R*_*o*_ − 1)*γ*, where *R*_*o*_ = 4.5, and *γ* = (1/14)*d*^−1^. Negative deviations from the dashed line reflect deviations from exponential in this phase-space representation.

**Fig 4:**
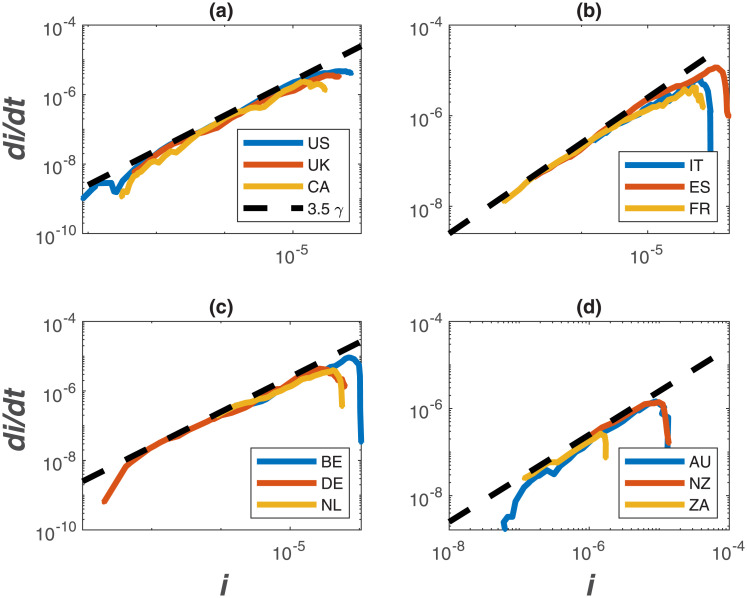
Same as Fig 3 but for sample countries. (a) the United States of America (US), the United Kingdom (UK), and Canada (CA); (b) Italy (IT), Spain (ES), and France (FR); (c) Belgium (BE), Germany (DE), the Netherlands (NL); (d) Australia (AU), New Zealand (NZ), and South Africa (ZA).

The same log-log scheme featured in Fig 2d is now applied to the global data set supplied by the European Center for Disease Prevention and Control (ECDPC). The data source provides daily confirmed infections *I*(*t*) and deaths reported for each country. The population of each country, used to estimate *S*_*o*_ (i.e. all members are susceptible), was obtained from the 2018 United Nations census and provided as part of the ECDPC data base. While daily data are supplied, not all countries report consistently on a daily *I*(*t*). For this reason, daily data on infections were smoothed with a 7 day block-average and *dI*/*dt* was estimated from the smoothed data. The 7-day block smooths out some of the spurious reporting during certain time periods (e.g. over weekends or during days when the health-care system was overwhelmed and processing along with reporting delayed by few days). It is to be noted here that the abscissa and ordinate are normalized by the same country-level population, meaning that the actual magnitude of *S*_*o*_ is not essential. However, such normalization allows for country-to-country comparisons in the same phase-space. The results show a global convergence to *R*_*o*_ = 4.5 from early time-analysis in Fig 3. Examples for specific countries are also featured in Fig 4 illustrating the same early slope patterns. Mindful of all the pitfalls in determining *R*_*o*_ [17], the global estimate here of *R*_*o*_ = 4.5 is roughly commensurate with other entirely independent estimates for COVID-19 discussed in the appendix and featured in Fig 1. The most recent update from a China study suggests an *R*_*o*_ = 4.1 [30] whereas for France, the most recent estimate for early times is *R*_*o*_ = 4.9 [31]. The initially reported and the much cited *R*_*o*_ = 2.2 value [4] from Wuhan, China appears to be low [32] as already noted in Fig 1. A more elaborate estimate of *R*_*o*_ based on case reports, incubation periods, high-resolution real-time human travel data, infection data combined with agent-based mathematical models result in *R*_*o*_ = 4.7 − 6.6 [32]. Other studies report values between 3.3 and 6.6 [33]. It must be emphasized that the *R*_*o*_ determined here reflects ‘country-scale’ early times assuming the entire country population to be *S*_*o*_, *γ* = (1/14)*d*^−1^ and does not accommodate any early measures enacted to reduce *β* or increase *γ*, which were undertaken in China [13] and other countries (e.g. Germany).

### Sub-national dynamics and interventions

**Fig 5:**
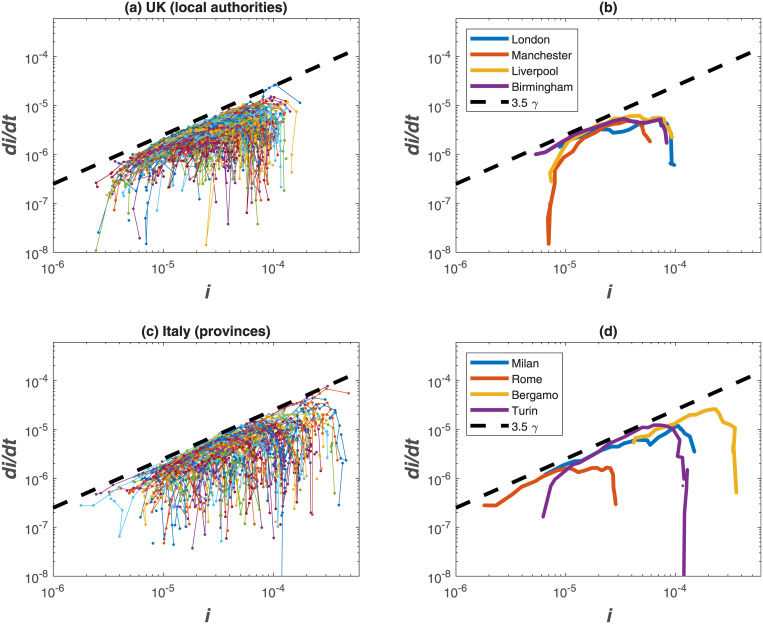
Same as Fig 3 but for sample UTLAs in the UK (a) and provinces in Italy (c). Selected UTLAs and provinces are shown in panels b and d, respectively.

The same analysis performed for World countries is now applied at a sub-national level, considering Upper Tier Local Authorities (UTLAs) in the UK and provinces in Italy (Fig 5). Results show a higher variability than country-level data (as expected) but the theoretical ‘boundary-line’ of *R*_*o*_ = 4.5 is shown to hold also at finer spatial scales. Cases reported at the beginning of April demonstrate that UK regions are at an early phase of the epidemics (with more ramp-up in testing as later discussed), while Italian provinces are approaching the peak of infections due to strict interventions put in place by national authorities.

Non-pharmaceutical interventions (e.g., social distancing, hand washing, universal masking) are the only measures currently available to limit the spread of COVID-19 [34], while contact tracing and isolation have been implemented to contain infected individuals [35]. Simulation results [35] showed that a COVID-19 outbreak can be controlled within 3 months if such strategies are put in place rapidly and effectively. This has been confirmed in another study [36] that showed that the containment measures employed in China and aimed at reducing human-to-human aerial transmission, succeeded in reducing the reproduction number to below unity within 30 days from implementation.

To consider the impact of interventions that reduce the infection rate over time and have direct effects on local scale dynamics [13], a time-varying *R*_*o*_, labelled as *R*_*o*,*d*_ (i.e. dynamic) can be implemented in the SIR model. A logistic function captures temporal patterns in *R*_*o*,*d*_, the effective reproduction number, consistent with those estimated for other outbreaks [36–39],

$$\begin{array}{c} R_{o,d}=R_{c}+\frac{R_{o}-R_{c}}{1+exp(k_{c}\left(t-t_{50}\right))}, \end{array}$$ (9)

where *R*_*c*_ is the controlled value of the dynamic *R*_*o*,*d*_, *k*_*c*_ is the steepness of the intervention curve, and *t*_50_ is the time when *R*_*o*,*d*_ = (*R*_*c*_ + *R*_*o*_)/2. It is assumed that, through interventions, the initial *R*_*o*_ = 4.5 is reduced to a controlled value of *R*_*c*_ = 1.1 after 2/*γ* days.

**Fig 6:**
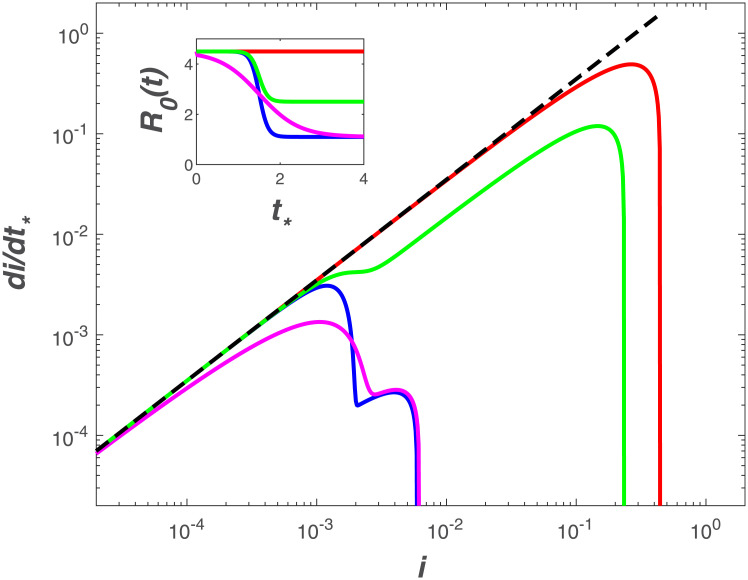
Modeled *di*/dt_*_ as a function of *i* when considering a dynamic *R*_*o*,*d*_. Five scenarios are illustrated (inset): no intervention (red) with *R*_*o*_ = 4.5 set to its uncontrolled value, *R*_*o*,*c*_ = 1.1 (epidemic near containment) and *k*_*c*_ = 0.7 (blue), *R*_*o*,*c*_ = 1.1 and *k*_*c*_ = 0.15 (magenta), *R*_*o*,*c*_ = 2.5 (typical of countries with strong initial intervention) and *k*_*c*_ = 0.7 (green). The other parameters of the logistic functions are *R*_*o*,*u*_ = 4.5 and *t*_50_ = 1.5/*γ*.

Model results accounting for different intervention scenarios (Fig 6) resemble the trends observed in the Italian provinces with the timing and magnitude of *R*_*o*_ reductions shifting the linear relation down and decreasing the maximum fraction of infected individuals. Such jumps are smoothed over at the national level where a clear deviation from exponential is observed (Fig 4).

### An alternative hypothesis: Power-law vs exponential

Whether these results are suggestive of a global convergence to an uncontrolled *R*_*o*_ = 4.5 or to some other dimensionless property must not be overlooked. A linear relation on a log-log representation may also be indicative of power-law solutions at early times, already documented in a number of studies for COVID-19 [40, 41]. In fact, published analysis of infection data from the top 25 affected countries reveals approximate power-law behavior of the form *I*(*t*) ∼ *t*^*a*^ (or log(*i*) = *a* log(*t*) + *b*) with two different growth patterns [40]: steady power law growth with moderate scaling exponents (i.e., *a* = 3-5) or explosive power law growth with dramatic scaling exponents (i.e., *a* = 8-11).

Within the confines of the SIR dynamical system framework here, we ask: what are the necessary modifications to obtain power-law solutions at early times? Such a solution, while not unique, may be possible by revising the force of infection as *βi*^*m*^. The original SIR model is recovered when *m* = 1. For this non-linear force of infection, the SIR system becomes

$$\frac{dS}{dt}=-\beta {\left(\frac{I}{S_{o}}\right)}^{m}S$$ (10)

$$\frac{dI}{dt}=+\beta {\left(\frac{I}{S_{o}}\right)}^{m}S-\gamma I$$ (11)

$$\begin{array}{cc} \frac{dR}{dt} & =+\gamma I. \end{array}$$ (12)

This revision ensures that the total population maintains its constant value (≈ *S*_*o*_ here). The early times dynamics (i.e. *S*(*t*) ≈ *S*_*o*_) for the non-dimensional infection compartment *i* are now governed by

$$\frac{di}{dt_{*}}=+R_{o}{\left(i\right)}^{m}-i.$$ (13)

When *m* < 1, maintaining a definition of *R*_*o*_ > 1 (epidemic), and noting that *i* ≪ 1, the first term on the right-hand side of Eq (13) is much larger than the second term. In fact, to obtain a maximum exponent enveloping the early-time relation between *di*/dt_*_ and *i*, the linear term can be dropped so that *di*/dt_*_ ≈ *R*_*o*_ *i*^*m*^ (only a growth phase). On a log-log representation, log(*di*/dt_*_) = *m* log(*i*) + log(*R*_*o*_). A constant slope such as those featured in Figs 3 and 4 may simply be estimates of *m* (instead of *R*_*o*_). The initial conjecture is that a power-law solution emerges from the modified SIR dynamics when *m* < 1. However, the slope here (= 3.5) actually exceeds unity contradicting this revised analysis. This finding supports the view that a global convergence to an uncontrolled *R*_*o*_ = 4.5 is a more likely explanation than a power-law alternative arising from a non-linear force of infection. To be clear, there are other causes for power-law solutions (e.g. a stochastic *β* as discussed elsewhere [42]), but those fall outside the domain of deterministic SIR approaches adopted here. Nonetheless, and as a bridge between the studies reporting power-law growth in time for *i* and the modified SIR here, a relation between *m* and *a* is sought. The solution to Eq (13) can be expressed as

$$i(t)={\left[i{\left(0\right)}^{1-m}+\beta (1-m)t\right]}^{1/(1-m)},$$ (14)

which is a power-law in *t*. For dimensionless time * γt* >> *i*(0)^1−*m*^/[*R*_*o*_(1 − *m*)], *i*(*t*) ∼ *t*^1/(1−*m*)^ (*m* < 1). It directly follows that *m* = (*a* − 1)/*a* < 1 (as expected), where *a* > 1 is determined by regressing early-times log(*i*) versus log(*t*). Reported *a* for what has been termed as ‘explosive’ cases such as the US, UK Canada, Russia, among others [40] all yield an *a* > 8 (with the US *a* > 16). Such high *a* simply confirms that *m* ≈ 1 (and without much variations), and the early time SIR dynamics does describe reasonably those cases. For low *a* values, termed as ‘steady’, the mean *a* ≈ 4.8, and thus yields an *m* ≈ 0.8, still not too far from unity. The shortcoming of analyzing *I*(*t*) upon *t* is that absolute figures of *I*(*t*) are sensitive to increased COVID-19 testing in time, which is considered next.

### Impact of testing ramp-up

A further explanation of early-time deviation from the SIR model (noted in several data sets here) may be time-dependent ramp-up of testing, which reveals existing infections at a rate faster than the infection spread. This hypothesis can be implemented in the SIR model considering the temporal dynamics of the testing capacity, *f*. Data show that testing capacity rates of increase depend on the country and follow linear or saturating trends [43]. To model a testing capacity that starts small and saturates over time, we assumed the maximum fraction of individuals that can be tested is *f* = 1, tests are 100% true and evenly distributed across compartments, and testing capacity grows exponentially at a rate *k*, independent of *I*, giving, *f*(*t*) = 1 − exp(−*kt*). Therefore, the apparent number of infections, *i*_*a*_, initially grows according to the superposition of the infectious spread rate and testing capacity increase rate, i.e., exp[(*R*_0_ − 1 − *k*)*t*] and log(*di*_*a*_/*dt*) ∼ (*R*_0_ − 1 − *k*)log(*i*_*a*_). From the data [43], we estimate a typical value of *k* is 0.02 d^−1^, which is negligible when compared to *R*_*o*_ − 1. The small value of *k* relative to *R*_*o*_ − 1 indicates that the imprint of testing ramp-up likely does not strongly impact the observed early-time dynamics and the observed convergent slope remains a robust indicator of the early phases of virus dynamics.

### The role of super-spreaders on *R*_*o*_ estimates

For COVID-19, there is currently no general agreement on a precise definition of a super-spreader. In its broadest interpretation, a super-spreader has the propensity to infect a larger than average number of susceptible individuals. This definition does not have a unique link to a precise mechanism and appears to encompass biological, behavioral and environmental variables relevant to disease transmission. We consider a narrow view of super-spreaders as those infectious individuals with high mobility. Within this narrower scope, the concern is to assess how long-distance mobility of few infected individuals (i.e. super-spreaders) impacts the estimates of *R*_*o*_ when the phase-space analysis of early times dynamics is used. Because these infectious individuals are highly mobile and depending on the mobility network in each country or region, detailed country-by-country investigation is beyond the scope here. However, the mobility of these super-spreaders can impact the much discussed mass-action assumption in SIR and thus estimates of *R*_*o*_ from early times dynamics.

We address this effect using an idealized yet generic analysis similar to the ramp-up testing effect earlier discussed. To do so, a country is first divided into identical and equal sized regions each of area given by *dx* and *dy*. These regions are ‘isolated’ and experience their own identical SIR dynamics assuming the same *R*_*o*_ and *γ*. Few infectious individuals arrive into this country (treated as a lattice in an *x* − *y* plane) at random locations thereby initially infecting few regions. Area wise, under 0.01% of the country area experiences an infectious individual at *t* = 0. To amplify the role of super-spreaders, mobility is only allowed between regions by super-spreaders, whereas mass action is still assumed between *I* and *S* within each region. To allow for large mobility of these super-spreaders in the SIR analysis, the budget equation for *I* in each region is revised to become an integro-differential equation (IDE) given as [44]

$$\begin{array}{c} \frac{\partial I}{\partial t}=(\beta \frac{S}{N_{o}}-\gamma )[\left(1-\phi \right)I+\phi \int_{-\infty }^{+\infty }\int_{-\infty }^{+\infty }I\left(x^{\prime },y^{\prime },t\right)p\left(x-x^{\prime },y-y^{\prime }\right)dx^{\prime }dy^{\prime }], \end{array}$$ (15)

where all state variables now evolve in space (x, y) and time (t) so that *I*, *S*, and *R* represent *I*(*x*, *y*, *t*), *S*(*x*, *y*, *t*), and *R*(*x*, *y*, *t*) (unless otherwise stated), *N*_*o*_ is the initial population in *dx* by *dy* region (*S*_*o*_ is the entire country population), *ϕ*(<< 1) is the fraction of infectious individuals that are mobile in a region within a given time step *dt* (super-spreaders of the *I* budget) and *p*(*x*′, *y*′) describes their spread kernel defined by the probability that infectious individuals at position *x*, *y* move to position *x*′, *y*′ in a time increment *dt*. The spread kernel must satisfy the normalizing condition

$$\begin{array}{c} \int_{-\infty }^{+\infty }\int_{-\infty }^{+\infty }p\left(x^{\prime },y^{\prime }\right)dx^{\prime }dy^{\prime }=1. \end{array}$$ (16)

When *ϕ* = 0 (no super-spreaders), the IDE approach reduces to spatially independent or autonomous SIR models operating in compartments *dx* × *dy* with no connectivity or spatial interaction between compartments (i.e. the entire country comprised of regions will experience the same *R*_*o*_).

A number of choices can be made about *p*(*x*′, *y*′), which all depend on the mobility network in each country (airports, roads, trains, public-transport, etc.) and analyzing all of them is beyond the scope here. For simplicity, we selected a distance-dependent spatial spread kernel [45]

$$p(r)=\alpha_{N}\text{exp}[-\frac{1}{2}{\left(\frac{r}{\sigma }\right)}^{2}].$$ (17)

where *r*^2^ = (*x* − *x*_*o*_)^2^ + (*y* − *y*_*o*_)^2^, *σ* is a measure of the spread of the spatial kernel, and *α*_*N*_ is a normalizing constant. Since the interest here is in spatial spread kernels with finite support *Ra* (i.e. super-spreaders cannot travel to all the corners of the domain in a single *dt*), *α*_*N*_ is determined so that

$$\begin{array}{c} \int_{r=0}^{r=Ra}p\left(r\right)=\alpha_{N}\int_{r=0}^{r=Ra}\text{exp}[-\frac{1}{2}{\left(\frac{r}{\sigma }\right)}^{2}]dr=1. \end{array}$$ (18)

This condition yields [45]

$$\alpha_{N}(Ra,\sigma )=\sqrt{\frac{2}{\pi }}\frac{1}{\sigma }{\left[\text{erf}(\frac{\text{Ra}}{\sqrt{2\sigma }})\right]}^{-1}.$$ (19)

**Fig 7:**
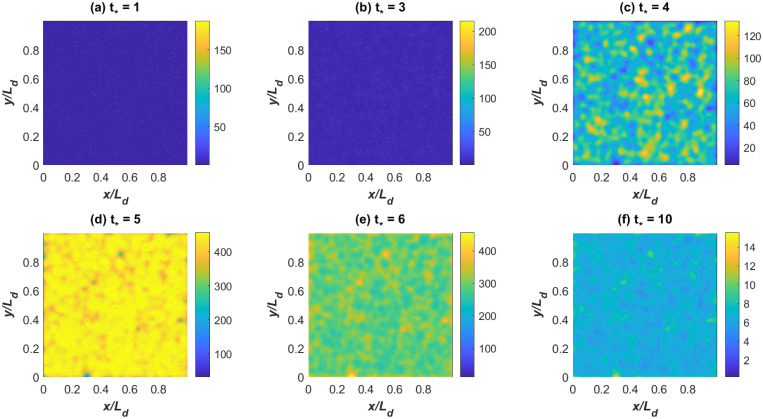
SIR model with super-spreaders. Modeled *I*(*x*, *y*, *t*) at selected t_*_ = *γt* showing the progression of disease outbreak in space due to mobility of super-spreaders only.

**Fig 8:**
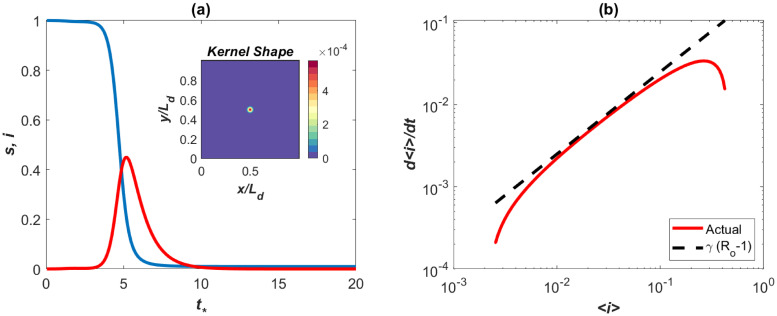
SIR model with super-spreaders: spatially integrated results. (a) Modeled < *s*(*t*) > and < *i*(*t*) >, where < . > is spatially integrated quantities (the inset shows the Gaussian spatial spread kernel). (b) The early times dynamics showing the effects of super-spreaders in the phase of *d* < *i* >/*dt* versus < *i* >. The line (*R*_*o*_ − 1)*γ* with *R*_*o*_ = 4.5 is shown for reference. This effect is similar to those reported in the UK and Italy regions.

Other spatial kernels can be specified and subjected to the same normalizing conditions thereby making the IDE approach flexible in terms of choices about spatial spread of infectious individuals. Also, it is possible to include time dependency in the spreading properties, meaning *p*(*x*′, *y*′, *t*) changes in time through temporal variations in *Ra* or *σ* or both (e.g. to allow for diurnal variations in mobility habits). Model calculations using the Gaussian kernel in Fig 7 show that super-spreaders (*ϕ* = 0.001) will infect almost the entire domain by t_*_ = 5. The main difference between Fig 2 and the spatially aggregated SIR model in Fig 8 is an initial delay and the occurrence of a rapid rise resembling the data from the UK and Italy regions at early times. However, beyond this initial delay in spreading, the early times dynamics with super-spreaders and without them are commensurate. That is, the effects of super-spreaders resembles the impact of testing ramp-up earlier discussed on the phase-space of *di*/dt_*_ versus *i* but with delays.

### Size of the epidemic

The maximum infections *I*_*max*_ (where *dI*/*dt* = 0) can be derived as a function of *S*_*o*_ and *R*_*o*_ by first dividing the budgets of *dS*/*dt* and *dI*/*dt*, solving the resulting equation, and noting that *dI*/*dt* = 0 when *S*(*t*)/*S*_*o*_ = *γ*/*β* = 1/*R*_*o*_ at *I*_*max*_ to yield

$$\begin{array}{c} i_{max}=\frac{I_{max}}{S_{o}}=1-\frac{1}{R_{o}}[1+\log(R_{o})]. \end{array}$$ (20)

**Fig 9:**
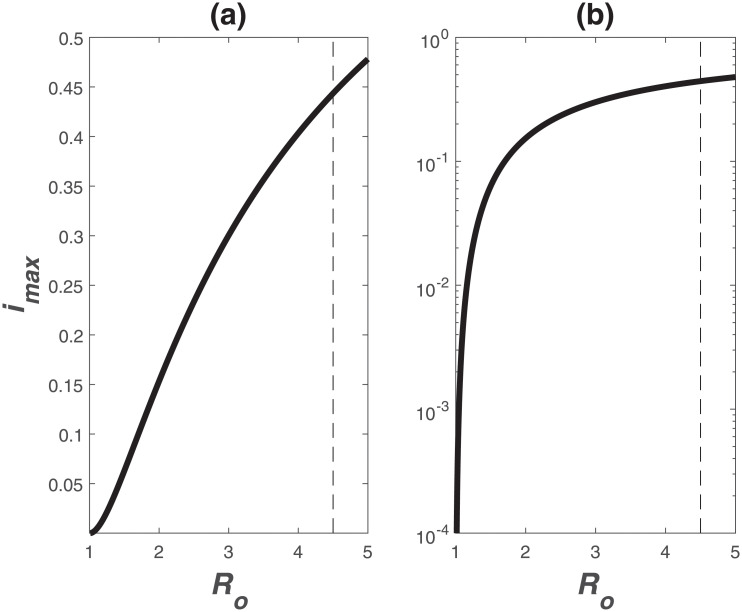
Relation between maximum infection fraction *i*_*max*_ = *I*_*max*_/*S*_*o*_ and *R*_*o*_.

Variations of *i*_*max*_ versus *R*_*o*_ are featured in Fig 9.

For *R*_*o*_ = 4.5, *i*_*max*_ = 0.44, which is much higher than values obtained for the common cold or the flu (*R*_*o*_ = 2 − 3) or influenza (*R*_*o*_ = 1.4 − 2.8).

The most significant use of *R*_*o*_ is an estimate of the size of the epidemic. The total fraction of infected individuals may be inferred from 1 − *S*(∞)/*S*(0), where *S*(∞)/*S*(0) = 1 − *R*(∞)/*S*(0) > 0 because *I*(∞) = 0. The relation between *S*(*t*) and *R*(*t*) can be derived

$$\begin{array}{c} \frac{dS}{dR}=-\frac{\beta }{\gamma }\frac{S}{S_{o}}, \end{array}$$ (21)

which when integrated between *t* = 0 and *t* = ∞ yields,

$$\begin{array}{c} \log[\frac{S(\infty )}{S_{o}}]=R_{o}(\frac{S(\infty )}{S_{o}}-1). \end{array}$$ (22)

**Fig 10:**
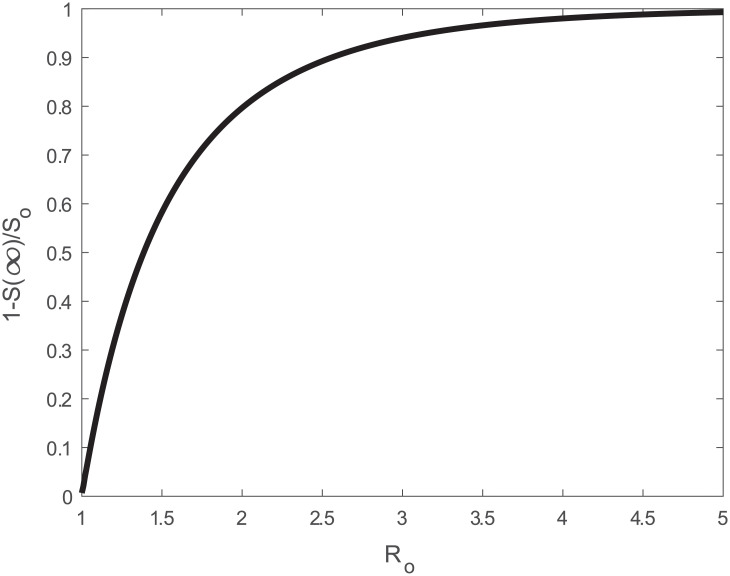
Relation between total infection fraction 1 − *S*(∞)/*S*_*o*_ and *R*_*o*_.

The solution for *S*(∞)/*S*_*o*_ can be analytically derived and linked to the total infected individuals *I*_*T*_ using

$$\begin{array}{c} \frac{I_{T}}{S_{o}}=1-\frac{S(\infty )}{S_{o}}=1+\frac{W[-R_{o}\exp\left(-R_{o}\right)]}{R_{o}}, \end{array}$$ (23)

where *W*[*z*] is the Lambert W-function of argument *z*. For pre-specified *R*_*o*_, the behavior of the *I*_*T*_/*S*_*o*_ individuals is shown in Fig 10. With such a high *R*_*o*_ = 4.5, some 98% of the population will be infected as t_*_ → ∞. When mortality is assumed to be some fraction of *I*_*T*_, then the mortality fraction is *M*_*o*_/*S*_*o*_ = *α*_*m*_[1−*S*(∞)/*S*(0)].

Different definitions of the global mortality rate *α*_*m*_ have been considered but, for the purposes of the present study, the ratio of deaths to both symptomatic and asymptomatic cases is most appropriate. This ratio is dubbed the Infection Fatality Rate (IFR) and is different from the Case Fatality Rate (CFR), which is the ratio of deaths to the number of confirmed cases. The IFR is more appropriate because the ‘Infected’ compartment of the SIR model comprises of both symptomatic and asymptomatic individuals. According to the latest IFR estimates, *α*_*m*_ ranges between 0.53 and 0.82% with a mean value of 0.68% [46].

**Fig 11:**
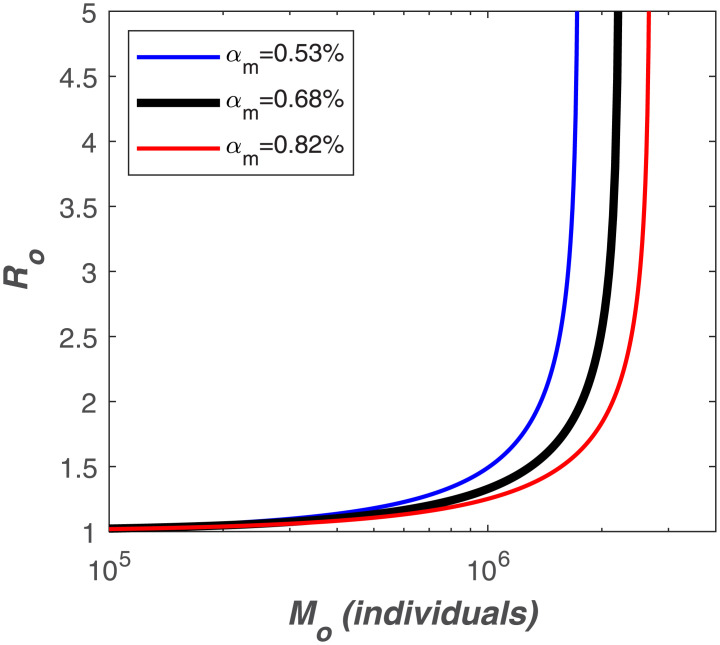
Relation between total mortality (*M*_*o*_) and *R*_*o*_ for different values of *α*_*m*_ and assuming *S*_*o*_ = 327M.

For the USA, the epicenter of COVID-19 at the time of submission of this article on April 10, 2020, we asked how much *R*_*o*_ should be reduced by deliberate intervention to maintain mortality below a certain threshold size *M*_*o*_. With *S*(0) = 327M, we determine how much *R*_*o*_ should be reduced as a function of *M*_*o*_ assuming different values of *α*_*m*_. These results are featured in Fig 11 and suggest that to maintain mortality below 1 million, *R*_*o*_ < 1.5 when *α*_*m*_ = 0.53%, a reduction factor of 3 over its uncontrolled value. Under the same scenario, to limit the death toll below 300 thousand people, *R*_*o*_ should be reduced to 1.1, a reduction factor of 4. These reduction factors are not entirely unreasonable using non-pharmaceutical measures (social distancing, masks, hand sanitizing, etc…).

**Fig 12:**
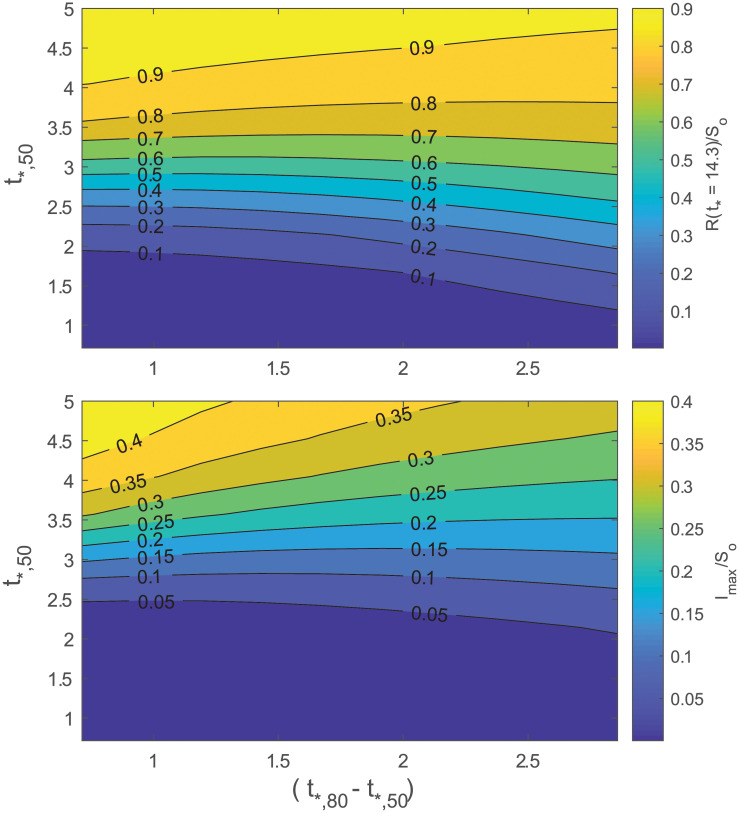
Variation in cumulative number of infected relative to *S*_*o*_ (top) and in maximum number of infected relative to *S*_*o*_ (bottom). The logistic form of *R*_*o*_ was used (Eq (9)). The *R*_*o*_ was set to vary from *R*_*o*,*c*_ = 4.5 to *R*_*o*,*u*_ = 1.0. The t_*,50_ and t_*,80_ are the dimensionless times at which *R*_*o*_ is half and 80% through the the total decline.

A natural extension of this exercise is to consider temporal changes in *R*_*o*_ following the logistic form in Eq (9). The maximum number of infected *I*_*max*_ at time *t* and cumulative number of infections *R*(∞) ≈ *R*(t_*_ ≈ 14) can be made to vary as the slope *k*_*c*_ and *t*_50_ are changed (Eq (9)). A larger *k*_*c*_ signifies more rapid enforcement of intervention policies and a larger *t*_50_ represents later enforcement. To provide a physical meaning for *k*_*c*_, we define $t_{f_{o}}$ as the time after first infection at which *R*_*o*_ is *f*_*o*_% of the way through its total decline from *R*_*o*,*u*_ to *R*_*o*,*c*_. With these definitions, $t_{f_{o}}=t_{50}+\log\left[f_{o}/\left(1-f_{o}\right)\right]/k_{c}$. An obvious choice for *R*_*o*,*u*_ = 4.5, the global average when no intervention is enforced. A logical choice for *f*_*o*_ = 80% and is consistent with the point at which the logistic function enters the ‘flattening phase’. We choose *R*_*o*,*c*_ = 1.0 to represent the most optimistic scenario of a near-containment by non-pharmaceutical intervention. For reference, the South Korea data suggests that early intervention, even when rapidly enforced shortly after the outbreak, resulted in *R*_*o*_ = 1.5 [47]. The effectiveness of interventions and any delays can now be linked to mortality and severity by varying *t*_50_ and *t*_80_. Fig 12 presents how *R*(t_*_ ≈ 14) and *I*_*max*_ are contained for only a restricted envelope of speed and timeliness of policy enforcement. The *R*(t_*_ ≈ 14) represents the cumulative number of fatalities and *I*_*max*_ is proportional to the degree to which resources, like hospital intensive care units, will be overwhelmed at peak infection rate. The results in Fig 12 indicate that if *R*_*o*_ > 2.7 within *t* = 3.5/*γ* (about 49 d here), a more than 10% reduction relative to *S*_*o*_ in *I*_*max*_ or *R*(∞) is unlikely.

An implication of Fig 12 is that if *R*_*o*_ does not decrease to at least 2.7 by 44 days after first infection, more than a million people are expected to die with an assumed constant mortality rate of 0.53%. For mortality to be confined to a range in the 100,000, then a reduction of *R*_*o*_ from 4.5 to 2.7 must be achieved within 14 days of first infection, which did not occur in the USA. As of July 30, 2020, confirmed fatalities in the USA have exceeded 150,000.

Last, it is to be noted that the fraction of individuals that must be immune (either through vaccination or recovery from prior COVID-19 infections) must exceed the herd immune threshold (HIT), which is given by

$$\begin{array}{c} p_{c}=1-\frac{1}{R_{o}}=0.78. \end{array}$$ (24)

This estimate of HIT sets the limit on the immune population needed to overcome another COVID-19 pandemic (assuming a global constant *R*_*o*_ = 4.5 and no intervention). Should immunity from prior COVID-19 infections be transient, this estimate then sets the upper bound on the fraction of population that must be vaccinated and the vaccine needed in the future.

## Conclusion

The work here has shown a global convergence of *R*_*o*_ = 4.5 when no deliberate intervention was taken for COVID-19. This *R*_*o*_ was shown to describe reasonably the maximum initial exponential growth rate of COVID-19 (=(*R*_*o*_ − 1)*γ*, where *γ* = (1/14)*d*^−1^) in many countries that did not initiate preventive measures within *γt* = 2. The findings here further support the growing consensus that the initial *R*_*o*_ = 2.2 estimate from Wuhan, China is low. The value of *R*_*o*_ = 4.5 is much more in line with other estimates (*R*_*o*_ = 4 − 6) derived from far more complex models. Model calculations and theoretical considerations offered here delineate the conditions when this *R*_*o*_ estimate is robust to the inclusion of other mechanisms such as super-spreaders and ramp-up in initial testing. The critical herd immunity level that must be reached is 78% to ensure COVID-19 does not become an epidemic again. This estimate sets a maximum limit on the vaccination required.

## Appendix: Compilation of *R*_*o*_ estimates from previous studies

A recent review [21] showed that existing estimates of *R*_*o*_ range from 1.4 to 6.49, with a mean of 3.28 and an interquartile range of 1.16. Early studies reported lower *R*_*o*_ values [21] and results often vary with the estimation method. Stochastic, statistical, and mathematical methods provided mean estimates of 2.44, 2.67, and 4.2, respectively [21], and SIR-based approaches are likely to provide higher values compared to other methods [36]. Similar results were presented in another meta-analysis [48] reporting a mean *R*_0_ of 3.38 (values ranging between 1.90 to 6.49) but did not find a significant effect of different estimation methods. A summary of these results is provided in Table 1.

**Table 1:** Compilation of published *R*_*o*_ estimates.

Reference	*R*_0_	*R*_0_ range	Study date
		(e.g. 95% CI)	(from-to)
Sanche et al. [22]	5.7	3.8—8.9	15 Jan. 2020—30 Jan. 2020
Li et al. [4]	2.2	1.4—3.9	N/A—22 Jan. 2020
Wu et al. [49]	2.68	2.47- 2.86	31 Dec. 2019—28 Jan. 2020
Riou and Althaus [50]	2.2	1.4—3.8	N/A—18 Jan. 2020
Liu et al. [39]	4.5	4.4—4.6	N/A—07 Feb. 2020
Zhang et al. [51]	5.6	5.3—5.8	21 Jan. 2020—20 Feb. 2020
Shen et al. [52]	4.7	4.50—4.92	12 Dec. 2019—22 Jan. 2020
Liu et al. [53]	2.90	2.32—3.63	N/A—23 Jan. 2020
Liu et al. [53]	2.92	2.28—3.67	N/A—23 Jan. 2020
Read et al. [54]	3.11	2.39—4.13	01 Jan. 2020—22 Jan. 2020
Majumder and Mandl [55]	2.55	2.0—3.1	08 Dec. 2019—26 Jan. 2020
WHO (from [21])	1.95	1.4—2.5	N/A—18 Jan. 2020
Cao et al. [30]	4.08	3.37—4.77	N/A—23 Jan. 2020
Zhao et al. [56]	2.24	1.96—2.55	10 Jan. 2020—24 Jan. 2020
Zhao et al. [56]	3.58	2.89—4.39	10 Jan. 2020—24 Jan. 2020
Imai et al. [57]	2.6	1.5—3.5	N/A—18 Jan. 2020
Tang et al. [58]	6.47	5.71—7.23	N/A—22 Jan. 2020
